# The Traumatic Brain Injury-Alzheimer’s Disease and Alzheimer’s Disease-related Dementia Caregiver Support Intervention: A Mixed Methods Evaluation of Program Feasibility, Acceptability, and Utility

**DOI:** 10.1093/geroni/igaf057

**Published:** 2025-06-18

**Authors:** Mara Wilson, Robyn W Birkeland, Elizabeth Albers, Katie W Louwagie, Sherry S Chesak, Edward Ratner, Jacob Finn, Samantha Ostenso, Joseph E Gaugler

**Affiliations:** School of Public Health, University of Minnesota, Minneapolis, USA; School of Public Health, University of Minnesota, Minneapolis, USA; School of Public Health, University of Minnesota, Minneapolis, USA; School of Public Health, University of Minnesota, Minneapolis, USA; Division of Nursing Research, Mayo Clinic, Rochester, USA; School of Nursing, University of Minnesota, Minneapolis, USA; Geriatric Research Education and Clinical Center, VA Medical Center, Minneapolis, USA; Medical School, University of Minnesota, Minneapolis, USA; Rehabilitation & Extended Care, Minneapolis VA Health Care System, Minneapolis, USA; Psychiatry & Behavioral Sciences, University of Minnesota, Minneapolis, USA; School of Public Health, University of Minnesota, Minneapolis, USA; School of Public Health, University of Minnesota, Minneapolis, USA

**Keywords:** Caregiving, Dementia, Psychosocial, Psychoeducational

## Abstract

**Background and Objectives:**

Research has established that unpaid family members, friends, or others who care for persons with dementia (ie, caregivers) may encounter socioemotional and physical health concerns as a consequence of providing extensive assistance. Similarly, caregivers for people living with traumatic brain injury (TBI) often experience a range of stressors and negative mental health outcomes due to care demands. Individuals with TBI often develop Alzheimer’s disease and Alzheimer’s disease-related dementia (AD/ADRD). This history of TBI may introduce complications to AD/ADRD caregiving. A comprehensive intervention grounded in the understanding of the complex caregiving context of both diagnoses is warranted to address the unique needs and concerns of TBI-AD/ADRD caregivers.

**Research Design and Methods:**

This study evaluated the feasibility of the TBI-AD/ADRD Caregiver Support Intervention (TACSI) psychoeducational program, designed to support the unique subset of caregivers facing the challenge of assisting relatives with AD/ADRD and a history of TBI. TACSI, a 6-session telehealth intervention, provides tailored psychosocial and psychoeducational coaching. In partnership with the 2 national healthcare centers, 15 caregivers enrolled in the 3-month feasibility study evaluating the design and subsequent refinement of TACSI. Mixed methods data were collected from 3-month follow-up surveys and qualitative data from postintervention interviews.

**Results:**

The feasibility, utility, and acceptance of TACSI were established. Caregivers liked the telehealth delivery and the personalized nature of TACSI, yet some expressed it would have been more beneficial earlier in their caregiving journey.

**Discussion and Implications:**

Caregivers valued the TACSI program. Their feedback has been applied to improve TACSI content and delivery for a larger pilot randomized controlled trial that is currently underway.

**Translational Significance:** We developed this study to evaluate the feasibility of TACSI to address the complications that a history of TBI may introduce to AD/ADRD caregiving. The study established the feasibility, utility, and acceptance of TACSI. These findings highlight this intervention’s significance in addressing the unique needs and concerns of TBI-AD/ADRD caregivers.

## Background and Objectives

Family and friends provide approximately 75% of care for individuals with Alzheimer’s disease and Alzheimer’s disease-related dementias (AD/ADRD) (1). Dementia caregivers often experience adverse impacts stemming from care provision, such as higher levels of stress, burnout, and declines in physical health (2,3). Approximately one-third of dementia caregivers report symptoms of anxiety and depression, while they also report greater use of negative coping strategies compared to their noncaregiving peers (4,5).

Similarly, family and friends are responsible for most of the care provision for individuals with traumatic brain injury (TBI) (6,7). Due to the impact on their cognitive and/or physical abilities, individuals with TBI frequently are highly dependent on their families, who are often unexpectedly thrust into the role of providing constant care (8). Studies suggest that TBI caregivers can experience significant burden and poor quality of life (9–11). This chronic stress can negatively affect their physical health (8).

Traumatic brain injury is a significant risk factor for dementia (12). Traumatic brain injury is associated with a 1.68 times greater risk of dementia in a 5-year cohort study (13). Female veterans with military-related TBI were found to have a 50%–80% increase in developing dementia (14). With the frequent co-occurrence of these diagnoses, there is a significant subset of caregivers who are navigating care-related stressors for their relatives’ intense needs. Research indicates that these caregivers of individuals with AD/ADRD and TBI often experience a higher degree of stress and burden compared to caregivers of those with physical disabilities (15).

A variety of interventions have been developed to address the complex challenges of dementia caregiving. Studies have targeted factors associated with increased burden, including avoidant coping styles, time spent caregiving, financial strain, and behavioral challenges or care needs (16–18). The interventions typically offer psychosocial support, respite, or skills training through support groups, psychotherapy, psychoeducation, and case management (19). These interventions are most effective when caregivers actively participate in programs tailored to their individualized needs with content that addresses the needs of the caregiver and person with AD/ADRD jointly (20,21). In addition, interventions have been shown to reduce caregiver depression (22), anger, fatigue (23), and physical morbidity (24), as well as improve overall quality of life (25).

Although the dementia care intervention literature continues to grow, there are fewer TBI-support interventions for caregivers. The Department of Veterans Affairs, in recognition of this lack of TBI-support, sought to address TBI caregiver needs with its Program of Comprehensive Assistance for Family Caregivers, which has demonstrated increased utilization of available supports for patients and caregivers (26). The limited existing TBI-support interventions tend to utilize similar methodologies as known dementia care interventions, focusing on providing support and/or skill building to reduce burden and depressive symptoms and improve coping strategies (27–29). A recent systematic review of TBI caregiver interventions found that of the 10 randomized controlled trials (RCTs) identified, the most effective interventions were tailored and caregiver-specific, employing a combination of psychoeducation and skills building (30). The TBI literature emphasizes that additional evidence-based caregiver support interventions should include accessible, comprehensive education and emotional support not limited by geography (31).

Previous interventions only targeted either TBI or AD/ADRD caregiving, focusing on the functional limitations (9) and caregiving issues specific to these disease contexts. However, individuals with TBI often develop AD/ADRD. This TBI history introduces potential complications to AD/ADRD caregiving, including any remaining TBI concerns (physical, psychological, behavioral), the impact of TBI on the presentation of dementia, and a potential need to modify previously established caregiving routines to accommodate the complexity of the dual diagnoses present. For example, individuals with TBI have a risk of greater functional decline in AD (32) as well as earlier onset (33). They are also more likely to have an increased prevalence of disordered mood and behavior symptoms such as depression, agitation, irritability, and greater levels of disinhibition as well as physical symptoms of motor dysfunction, including gait disorders and falls, when compared to individuals with AD/ADRD alone (34). As such, the development and evaluation of a comprehensive intervention grounded in the understanding of the unique needs of those caring for individuals with both TBI and AD/ADRD is warranted.

The TBI-AD/ADRD Caregiver Support Intervention (TACSI) is a novel intervention that provides one-on-one telehealth support to caregivers of individuals with a dual diagnosis of dementia and a history of TBI. The TBI-AD/ADRD Caregiver Support Intervention combines best practices from AD/ADRD and TBI caregiving literature to provide a tailored coaching model that aims to improve TBI-AD/ADRD caregivers’ coping strategies and increase their awareness of socioemotional support (see Supplementary Table 1 for program objectives and key activities). The TBI-AD/ADRD Caregiver Support Intervention offers psychoeducation to build skills and strengthen coping strategies for TBI and AD/ADRD concerns (eg, active coping and planning; emotion-focused coping) and promotes awareness and availability of social support (eg, emotional, instrumental). With consideration for both diagnoses, TACSI was designed to positively influence key outcome domains for caregivers: primary subjective stress, secondary role strain, care-related distress, and depressive symptoms.

The program utilizes the Stress Process Model (SPM) as its guiding conceptual framework (35). The SPM has served as a comprehensive framework for family caregiver interventions across disease contexts, including AD/ADRD and TBI (36–39). The model asserts that as care demands intensify (eg, memory impairments, functional declines, behavioral disruptions; ie, primary objective stressors), caregivers are at greater risk for experiencing emotional distress (eg, feelings of emotional exhaustion, being trapped in the caregiving role; ie, primary subjective stressors) (35). The more care-related stress, the more likely caregivers are to experience negative mental and physical health ramifications.

This preliminary evaluation of TACSI utilized quantitative and qualitative mixed methods to determine how caregivers of individuals with TBI and AD/ADRD perceived the intervention’s utility, acceptability, and feasibility.

## Research Design and Methods

### Recruitment and Design

The University of Minnesota School of Public Health (UMN) collaborated with Mayo Clinic—Rochester and the Minneapolis Veterans Administration Health Center’s (Minneapolis VA) Geriatrics Research Education Clinical Center to recruit caregivers of individuals with dual diagnoses of TBI and dementia. The Minneapolis VA’s Geriatrics Research Education Clinical Center is a national center with a research and clinical focus on dementia, and 1 of only 5 nationwide polytrauma centers with a TBI-specific program. Mayo Clinic has 2 nationally recognized research centers: the Alzheimer’s Disease Research Center and the Traumatic Brain Injury Model System. The UMN provided research project conceptualization and leadership, coordination, intervention delivery, data management, and analysis. The UMN and Mayo Clinic obtained a single Institutional Review Board approval (STUDY00014886). The Minneapolis VA required site-specific approval (1641329).

International Classification of Diseases (ICD) codes from patient records were utilized to compile lists of eligible parties at each site (Mayo Clinic—Rochester and the Minneapolis VA). Eligible codes included dual diagnoses of G30.9 (Alzheimer’s disease, unspecified), F03 (Unspecified dementia), S06.2 (Diffuse traumatic brain injury), and S06.3 (focal traumatic brain injury). Next of kin or emergency contacts were extracted for presumed caregivers’ contact information for patients with eligible dual diagnoses. Letters were mailed to caregivers or patients if a caregiver was not listed. One to four weeks after the letters, healthcare facility staff called potential participants to discuss the study. Mayo Clinic and the Minneapolis VA mailed 53 letters each, making follow-up calls to all with available phone numbers. Caregivers from the Mayo Clinic could contact the UMN directly or give their verbal or written permission to be contacted by the UMN. In contrast, caregivers from the Minneapolis VA were required to contact the UMN directly due to privacy regulations. The study was also posted on ClinicalTrials.gov and StudyFinder.umn.edu. UMN attempted to contact potential participants once a week for up to 4 weeks to complete enrollment.

Caregivers were eligible if their care recipient was diagnosed with TBI and AD/ADRD or unspecified dementia (based on the ICD-10 codes and/or caregiver confirmation) and they: (a) identified as providing the most help or being most responsible for the person with TBI-AD/ADRD (could share the role equally with another caregiver); (b) indicated willingness to participate in the TACSI evaluation; (c) were English-speaking; (d) were 21 years or older; (e) were not participating in other coaching or psychosocial consultation specific to caregiving; and (f) resided in the United States. Caregivers were not eligible if they had a worsening mental health condition and were not currently receiving treatment or were taking psychotropic medication(s) with a dosage that had not been stable at least 3 months.

Eligibility screening was conducted over the phone. Those recruited from the Minneapolis VA or Mayo Clinic did not need to confirm their care recipient’s TBI and AD/ADRD or cognitive impairment diagnoses, as the recruitment sample was identified based on ICD codes in medical records. Caregivers recruited from other sources were asked to confirm the presence of both diagnoses. The remaining screening criteria were applied in an identical fashion.

**Figure 1.**
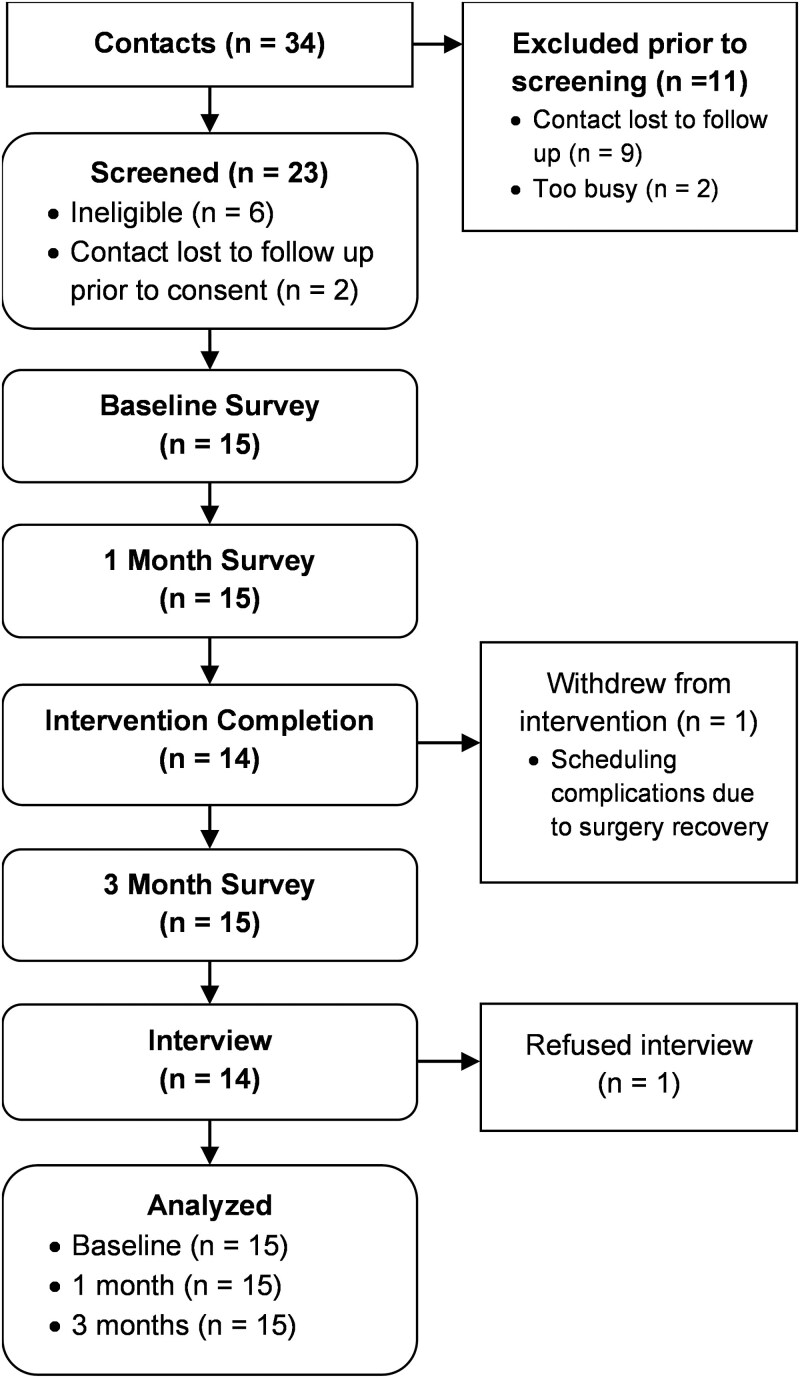
CONSORT flow diagram.

The UMN received 34 potential participant contacts. Most contacts reached out to the study team directly after receiving a call from their health system, and rarely after receiving the letter alone. Eleven potential participants were excluded prior to screening. Twenty-three completed the eligibility screening. Seventeen were deemed eligible, with 2 becoming lost to follow-up prior to providing consent. See the CONSORT diagram (Figure 1) for participant flow details. Individuals deemed not eligible were offered resources. Most caregivers provided consent via an online consent form (*n* = 12). In contrast, others read the consent form individually and provided verbal consent over the phone (*n* = 2) or read the consent form over the phone (*n* = 1).

Caregivers were sent baseline and 3-month follow-up surveys, which could be completed via mail, online, or phone. Baseline surveys included validated measures that have demonstrated strong psychometric properties in prior caregiving research. These measures evaluated care recipient behavior issues, ADL/IADLs, caregiver stress, depressive symptoms, well-being, self-efficacy, coping style, interpersonal support, and service use. See Supplementary Table 2 for measure descriptions, baseline Cronbach alphas, and references. Follow-up surveys added measures to determine TACSI’s: (a) acceptability with the 4-item Acceptability of Intervention Measure (AIM); (b) appropriateness with the 4-item Intervention Appropriateness Measure (IAM); and (c) feasibility with the 4-item Feasibility of Intervention Measure (FIM) (40). These measures are valid, have high internal consistency (AIM *α* = .85, IAM *α* = .91, and FIM *α* = .89), and have high test-retest reliability (.80, .73, .88, respectively). Items were scored on a 5-point scale (1 = completely disagree; 5 = completely agree).

A treatment receipt checklist (TRC) was adapted for this study from other dementia care interventions delivered by the UMN team (41–43). The TRC included 12 Likert scale items regarding specific intervention content, delivery, and willingness to recommend the intervention (1 = strongly disagree to 5 = strongly agree, with an option to indicate does not apply to me/my situation). The TRC concluded with 1 open-ended question asking how the intervention was or was not helpful. Semistructured interviews (SSI) were conducted by phone within 6 weeks of completing the 3-month follow-up survey. The interviews examined whether and how caregivers benefited from TACSI. See Supplementary Table 3 for the Interview Guide.

All 15 caregivers completed the baseline and 3-month follow-up surveys. Fourteen of 15 participated in the semistructured interviews, with 1 participant declining to participate. Refer to Figure 1 for details. The interviews were completed from November 2022 through June 2023. Caregivers were compensated $25 for each survey and interview completed, for up to $75 total.

### Intervention

The TBI-AD/ADRD Caregiver Support Intervention combines psychosocial and psychoeducational approaches to: (a) identify stressors related to caregiving for family members with a diagnosis of both TBI and AD/ADRD or unspecified dementia; and (b) support caregivers to develop more effective communication and coping strategies, along with improving caregiving self-efficacy. This 6-session semistructured intervention is tailored to address the individual needs of a family caregiver by focusing on their experiences adjusting to the dual diagnosis of TBI and dementia.

The intervention sessions are designed to support the caregiver’s acquisition of information and strategies to reduce stress and manage issues related to caregiving for someone with TBI and dementia. The content serves to address 6 key objectives: (a) increasing caregiver understanding of dementia and TBI; (b) reducing caregiver stress; (c) exploring and offering support for caregivers’ experiences of guilt and grief; (d) enhancing effective communication, particularly with family and working with healthcare teams; (e) increasing caregiver awareness of dementia and TBI supports and services; and (f) enhancing family support.

Through individual coaching and ad hoc support, TACSI offers knowledge and skills to help caregivers manage stress and improve their quality of life. Based on caregiver preference, the intervention is delivered in one-on-one sessions with a trained coach via telephone or secure video conferencing. Coaches were 2 UMN study team members with doctoral degrees: 1 in clinical psychology (PhD) and the other in nursing (DNP).

Individual session content and prompts are semistructured. While sessions 1 and 6 follow a similar structure/content for all participants, the order of module content presented in sessions 2–5 is tailored to caregiver priorities. All modules are offered to caregivers; however, caregivers choose the topics they want to engage in more thoroughly based on their needs. All sessions include a general check-in regarding any updates or questions/concerns. See Table 1 for details about the intervention’s foci.

**Table 1.** Intervention Overview

Session	Module Content	Focus
1	In-depth interview	Exploring caregiver’s experiences providing care for their relative
2–5^*^	Understanding dementia and traumatic brain injury (TBI); building awareness of supports and services	Psychoeducation on TBI and dementia; how it affects the brain, cognition, personality and behavior; common behaviors and management strategies, local and national supports and services
	Emotional health and well-being	Discussion and support of guilt and grief, understanding of stress, stress management strategies
	Realistic expectations and adjusting to lifestyle changes	Encouraging realistic expectations, promoting activity ideas, and exploring caregiving roles
	Working with a healthcare team; enhancing communication	Tips to work with healthcare team, 4 steps for conflict resolution
6	Review of intervention topics and discussion of wrap-up questions	Caregiver adjustment, challenges, and strengths

^*^The order of the psychoeducation/support modules is tailored to caregiver priorities.

Ad hoc support was available to all participants between sessions and after intervention completion. This support was offered via telehealth or email communications based on the participant’s preference. Ad hoc communications were personalized to address each participant’s unique concerns in a prompt manner. They also provided a means of maintaining support after intervention completion.

### Data Analysis

Using frequency tables and means, descriptive quantitative analyses were conducted to examine ratings of every statement in the treatment receipt checklist, which included AIM, IAM, FIM, and the 12 Likert-scale TRC questions.

All interviews were audio recorded and professionally transcribed. Transcripts were analyzed in NVivo 13 (44). Braun and Clarke (45) and Williams and Moser (46) guided the reflexive thematic analysis. Authors MW, RB, KL, and EA reviewed transcripts to familiarize themselves with the data, then created a codebook from content that emerged repeatedly in the open-ended data. Interviews were conducted individually, gradually changing the codebook based on group discussions until saturation was met and the codebook did not require further revisions. This evolution of the codebook was documented internally and, together with group discussions of the codes, allowed our coding assumptions to be critically examined. Once the codebook was finalized, all 14 interviews were coded separately by 2 staff members along with the open-ended question responses from the 15 treatment receipt checklists. Following coding, authors MW, RB, KL, and EA met multiple times to discuss how the codes should be clustered into larger categories and themes. These qualitative themes were analyzed in tandem with the descriptive quantitative results to better understand the intervention’s mechanisms of benefit and how it could be refined for a pilot RCT.

## Results

### Quantitative Results

#### Demographics

Most caregivers in the study were female (93%), White (87%, with 1 also reporting as Hispanic/Latino), and married (73%). Most were caring for a spouse or partner (60%), with the remainder caring for a parent (33%) or child (7%). Additional demographics for caregivers and care recipients are presented in Table 2.

**Table 2.** Baseline Demographics of Caregivers and Care Recipients

Characteristics	*N* (%)	*M* ± *SD*
*Caregiver Characteristics (N = 15)*		
Female	14 (93.3)	
Age, in years		62.4 ± 10.1
White (alone)	13 (86.7)	
Hispanic/Latino	1 (6.7)	
Married	11 (73.3)	
Earned bachelor’s degree or higher	6 (40.0)	
Minnesota resident	9 (60.0)	
Relationship to care recipient		
Spouse or partner	9 (60.0)	
Daughter or son	5 (33.3)	
Other	1 (6.7)	
Years caring for care recipient		12.8 ± 15.3
Caregiver aware of care recipient’s TBI	13 (86.7)	
Caregiver aware of care recipient’s dementia (unspecified) diagnosis, including mild cognitive impairment	12 (80.0)	
*Care recipient characteristics (N = 15)*		
Female	2 (13.3)	
Age, in years		76.3 ± 11.4
White (alone)	14 (93.3)	
Hispanic/Latino	0 (0.0)	
Married	10 (66.7)	
Earned bachelor’s degree or higher	5 (33.4)	
Is a veteran	10 (66.7)	
Lives in care facility	7 (46.7)	
Most severe TBI occurred within last 5 years	4 (26.7)	
If known by caregiver, duration in years since TBI diagnosis (range: 1–60)		21.3 ± 20.5
TBI during military service (presence of 1 or more)	3 (20.0)	
If known by caregiver, duration in years since dementia diagnosis (range: under a year-54)		9.2 ± 15.4

*Note*: TBI = traumatic brain injury.

#### Process data

Fifteen caregivers were administered the intervention. Fourteen completed all 6 intervention sessions (1 withdrew from the intervention after completing 2 sessions due to scheduling complications following a surgical procedure, as documented in Figure 1). Caregivers completed the intervention, on average, in 6 weeks and 6 days (range: 4 weeks and 1 day to 10 weeks and 5 days). Five caregivers (33%) engaged in sessions solely via video conferencing, 4 (27%) participated solely via telephone, and 3 (20%) used both modalities. Session length averaged 59 min (range: 30–80 min). See Supplementary Table 4 for additional information.

Thirteen of the 15 participants engaged in ad hoc support. Ad hoc communications most often involved resource provision and emotional support. Most participants engaged in ad hoc support via email communication (44 total emails). Additionally, 3 caregivers opted to participate in individualized ad hoc sessions personalized to meet their specific needs (combined total: 7 sessions), with 2 participating in 2 ad hoc sessions via phone and the third participating in 3 ad hoc sessions via videoconferencing. Ad hoc session length averaged 27 min (range: 15–60 min).

#### Acceptability, feasibility, and utility

Analyses of caregiver responses on the IAM, AIM, and FIM measures (40) indicated that TACSI was acceptable (mean = 4.43, *SD* = 0.73), appropriate (mean = 4.32, *SD* = 0.75), and feasible for caregivers (mean = 4.25, *SD* = 0.72). Table 3 includes individual item means. High intervention completion rates also support TACSI’s feasibility and acceptability.

**Table 3.** Means and Standard Deviations of Intervention Acceptability, Appropriateness, and Feasibility Measure Items

Measure items	*M* ± *SD*
Acceptability of Intervention (AIM)	
1. TACSI meets my approval	4.53 ± 0.74
2. TACSI is appealing to me	4.33 ± 0.82
3. I like TACSI	4.53 ± 0.74
4. I welcome TACSI	4.33 ± 0.82
Intervention Appropriateness Measure (IAM)	
1. TACSI seems fitting	4.20 ± 0.86
2. TACSI seems suitable	4.27 ± 0.80
3. TACSI seems applicable	4.47 ± 0.74
4. TACSI seems like a good match	4.33 ± 0.82
Feasibility of Intervention Measure (FIM)	
1. TACSI seems implementable	4.27 ± 0.80
2. TACSI seems possible	4.27 ± 0.70
3. TACSI seems doable	4.20 ± 0.77
4. TACSI seems easy to use	4.27 ± 0.70

*Notes*: TACSI = Traumatic Brain Injury—Alzheimer’s Disease and Alzheimer’s Disease Related Dementias Caregiver Support Intervention.

Caregiver responses on the treatment receipt checklist corroborated these findings as caregivers endorsed agreement or strong agreement with all 12 statements about delivery and receipt of TACSI (overall mean = 4.66, *SD* = .56). Notably, 12 of 15 caregivers (80%) endorsed their strong agreement and the remaining 3 endorsed agreement with “I would recommend TACSI to other caregivers of individuals with a history of TBI and dementia.” See Table 4 for means of individual treatment receipt checklist items.

**Table 4.** Means and Standard Deviations of Treatment Receipt Checklist Items

Treatment Receipt Checklist Items: The TACSI Program	*M* ± *SD*
1. Provided education on dementia and traumatic brain injury (TBI) processes that affect the brain, behavior, and personality	4.77 ± 0.44
2. Provided strategies and tools to help manage individual symptoms of dementia and TBI	4.77 ± 0.44
3. Explained how stress affects the mind and body	4.47 ± 0.74
4. Shared coping, stress management, and relaxation strategies	4.86 ± 0.36
5. Encouraged self-care	4.79 ± 0.43
6. Helped me to identify, process, and reframe feelings of guilt	4.50 ± 0.65
7. Explored different grief experiences and their effects on me and family	4.69 ± 0.63
8. Introduced the 4 Steps of Conflict Resolution	4.42 ± 0.51
9. Shared strategies for communicating with the healthcare team and family	4.50 ± 0.65
10. Provided information on dementia and/or TBI support or services	4.64 ± 0.50
11. Explored the impact of dementia and TBI on family	4.67 ± 0.72
12. I would recommend TACSI to other caregivers of individuals with a history of TBI and dementia	4.80 ± 0.41

*Notes*: TACSI = the Traumatic Brain Injury—Alzheimer’s Disease and Alzheimer’s Disease Related Dementias Caregiver Support Intervention.

Of note, there were no significant differences in overall IAM (*F*(1,13) = .34, *p* = .57), AIM (*F*(1,13) = 0.66, *p* = .80), and FIM (*F*(1,13) = .19, *p* = .67) measures nor overall treatment receipt checklist scores (*F*(1,13) = .016, *p* = .90) between caregivers who participated in ad hoc sessions and those who did not engage in additional sessions.

### Qualitative Results

Utilizing caregiver interviews and the responses to the open-ended item on the treatment receipt checklist, mechanisms of benefit were identified. These mechanisms of benefit are related to aspects of the intervention’s delivery and its content. Subthemes are described below. Pseudonyms were given to safeguard caregiver confidentiality.

#### Intervention delivery

##### Session logistics

Several caregivers emphasized the benefit of one-on-one sessions. Meryl (spouse, interview) succinctly stated, “Just a one-on-one was really awesome.” Mary (spouse, interview) explained how she valued being able to focus on the topics she wanted, in contrast to participating in a support group where “lots of people would rather tell their stories, and I want information…The [interventionist] was very much an information provider…I really appreciated that.”

Most caregivers felt the 6-session intervention length was appropriate. Many expressed appreciation that ad hoc coaching was available if needed, although only 20% of caregivers utilized ad hoc sessions. Linda (spouse, interview) stated, ‘And [my coach] would say too “If you need to call me before [a scheduled session], feel free to do that.” Pam (spouse, interview) shared a similar sentiment, “I was kind of sad when it ended. But yet even with that I was still able to reach out to my counselor,” referring to the option for ad hoc consultation.

In addition, caregivers appreciated TACSI’s telehealth delivery. Shannon (spouse, interview), who met with her coach by phone, explained, “I think it was really good…even though I didn’t go in person.” Several caregivers also noted they valued not having to coordinate care for their care recipient to attend telehealth sessions.

##### Coach attributes

Caregivers indicated that they enjoyed talking with knowledgeable, caring, and nonjudgmental coaches. Sam (adult child, interview) valued having a coach “who is knowledgeable and could give clear direction or feedback.” Elena (adult child, interview) appreciated the “[coach] not only would either already have the answer or was more than willing to find it.” Caregivers described how a caring and nonjudgmental coach allowed them to explore and process their experiences in an accepting environment. Greta (spouse, interview) shared that “I didn’t feel any pressure…[and] that was really important to me, so I felt more free to talk, I felt more confident, and more trust to talk about stuff that I usually don’t talk about.” This open atmosphere allowed caregivers to feel heard and genuinely cared for. Caregivers valued sharing ideas and asking questions to an empathetic expert. Linda (spouse, interview) described how “there’s still some expectation [with others], like I need to be strong and get through this and whatever. And with [the coach], I can just let it all out.”

##### Brainstorming

Caregivers appreciated brainstorming with the coach about planning and guidance. Caregivers shared how problem-solving and talking through solutions helped reduce their stress. Pam (spouse, interview) reflected on her sessions with the coach, sharing, “And coming up with a plan…that created my ability to reach out to some friends in town.”

#### Intervention content: tailoring

##### Personalized session content

Caregivers valued the semistructured design of TACSI that allowed for individualized content and discussion of skills, strategies, and information. Providing a variety of self-care, communication, and behavioral management approaches to choose from helped caregivers to choose the strategy that best fits their unique situation. Sam (adult child, interview) described “...everyone’s needs are going to be different, and I can see how each of the topics that were given as an option would be relevant to different people in different situations. I think that the way it was presented to me set up my option of what I wanted to talk about, [was] the best way to do it.” Peggy (spouse, survey) agreed that “What works for one may not work for another.”

Shannon (spouse, interview) endorsed that coaches were responsive to providing content that fit the caregiver’s needs for each session instead of, “...This is what we’re going to talk about, whether you like it or not.” Elena (adult child, interview) added, “I think we fit everything in there that she needed to, but she was also there for those things that were just kind of consuming me in those moments.”

#### Intervention content: intervention strategies, information, and resources

##### Dementia and TBI education

Caregivers valued the TACSI program education surrounding dementia and TBI. Helen, (spouse, interview) shared, “I learned a lot from [the program], and it really supported me more than I thought I needed.” Chris (adult child, survey) noted, “Before TACSI I was struggling to understand dementia and how it affected mom.” Specifically, caregivers appreciated education related to behavior and communication, planning, and safety.

##### Behavior management and communication with care recipient

Program education seemed to facilitate caregivers’ interaction with their care recipient. Angela (adult child, interview) shared: “Maybe the TACSI study kind of made me look at things a little bit different…[care recipient’s] brain has had some changes and facts get all tangled up...So a lot of times we just listen now and let them talk.”

Helen (spouse, interview) further highlighted improved interactions with her care recipient as a result of her increased understanding and subsequent shift in perspective, “I don’t think I would’ve let go with wanting [care recipient] to see things exactly as I want him to see it if it weren’t for this program…[instead of telling him] ‘This didn’t happen’ or ‘That didn’t happen,’ looking at what the bigger picture is and not getting into those arguments.” She went on to describe that, “being more educated and learning communication things that are helpful or not helpful has benefitted [him].”

##### Future planning

Education about the dementia and TBI disease process influenced caregivers’ planning ability. Becky (adult child, interview) shared, “I’ll know what to expect and what’s gonna come next.” Caregivers valued learning more about available resources and discussing when to initiate these services and supports in the future. Caregivers attributed many changes to TACSI, including modifying care plans and instituting a power of attorney.

##### Safety

Several caregivers highlighted the impact of discussions around safety, including home safety and protection of financial information. Simone (adult child, interview) reflected on how the care recipient would behave dangerously, such as putting the coffee pot on the stove to make coffee, and “[Interventionist] helped me about that kind of stuff, and it’s going a lot better now.” In addition, TACSI helped caregivers to plan for and safely and effectively navigate healthcare encounters, including creating lists of medications and diagnoses to bring to medical appointments.

##### Communication skills and strategies with family and healthcare team

Caregivers emphasized the value of communication skills shared in TACSI, such as conflict resolution and rehearsal of important conversations. Caregivers valued how TACSI encouraged them to initiate open conversations with family members and increased their comfort in asking for and accepting help. Elena (adult child, interview), who had been reluctant to talk to her family about their father’s care, shared, “If I wouldn’t have been talking to [coach], I don’t think I would have ever reached out to them…and I’m glad that I did.” Meryl (spouse, interview) shared how TACSI not only helped secure more family involvement but also initiated some important family discussions around serious care issues: “Well, one really huge benefit was I asked my children to become involved, and shared some information with them, and asked their thoughts on short term, long term, if something would happen to me, if…something with their dad. That opened a door that I’ve kept closed forever, so that was really huge.”

Caregivers also felt TACSI gave them the skills and empowered them to address concerns with their care recipient’s health care. Elena (adult child, interview) shared, “It gave me that confidence to just be like, ‘Okay, it’s time for an open dialog. I can’t worry about if they think I’m complaining.’”

##### Self-care

TACSI coaches emphasized self-care, providing caregivers with techniques to reduce stress and better care for their health and well-being, including practicing mindfulness, relaxation exercises, and utilizing health and well-being, including mindfulness, relaxation exercises, and respite care. These self-care-focused discussions were useful for many caregivers. Chris (adult child, interview) stated, “it’s not a stretch to say that I’m in a very healthy place now because of that conversation.” Simone (adult child, interview) shared how TACSI “…took a lot of the stress away. When I first started talking to [interventionist], I wasn’t sleeping at all, I wasn’t taking care of myself, and she had told me that self-care is really important...she taught me a lot of self-care techniques and the different breathing [exercises].”

Meryl (spouse, interview) extended relaxation strategies to her husband, sharing, “We talked about how to relax during stressful times, and I had my husband (care recipient) utilize that.” Of note, some caregivers were already aware of some techniques but appreciated the reminder to use them. For example, Elena (adult child, interview) explained how they “had therapy before so, I’m familiar with kind of the…let’s just say I had the tools in my bag, I just hadn’t opened the tool bag in a while.”

##### Resources provided

Caregivers valued working with coaches to identify their needs and that coaches searched for local or national services and supports to address those needs, including healthcare system services, respite care, and alternative transportation options. Pam (spouse, interview) shared how difficult it can be for overwhelmed caregivers to initiate finding resources, “It’s just too much. So, having someone there in your corner helping you find those resources so all you have to do is call…That is a big bonus for the program.”

#### Recommendations for pilot RCT

##### Caregiver feedback

Caregiver recommendations from this feasibility study were incorporated into our current pilot RCT, when appropriate and applicable. Surveys were modified to accommodate caregivers whose care recipients lived in long-term care communities. Commonly requested or explored topics like lifestyle planning and balance, intimacy, and residential long-term care were added to the intervention manual.

Several caregivers who had been caregiving for many years shared that they would have benefited even more from the intervention had they been introduced to it earlier in their caregiving journey. For instance, Meryl (spouse, interview) expressed, “...had it been 10 years ago or 20 years ago, I think everything would have been something that I really could’ve utilized.” Sam (adult child, interview) said, “I thought it was really good,” adding that making it available “...more immediately, like once they find out that their family member needs help, that would probably help them a lot more.” Elena (adult child, interview) agreed, “…you all would have been a godsend to someone in the beginning stages of caring for a loved one like that.”

## Discussion and Implications

Caregivers of individuals living with TBI and AD/ADRD face a challenging life transition that requires them to address not only the physical needs of their care recipient but the emotional, spiritual, and economic effects of care provision as well. These needs are often not explicitly met by the healthcare system, leaving caregivers to navigate these issues on their own. As a result, caregivers frequently report high levels of stress and difficulty coping. With the number of individuals diagnosed with ADRD growing dramatically, support programs are essential to assist caregivers with navigating the considerable caregiving challenges of this dual diagnosis. While many programs focus on support for TBI or AD/ADRD or use a peer support model, the TACSI program is a comprehensive psychosocial and psychoeducational one-on-one coaching program uniquely tailored to support caregivers of individuals with a dual diagnosis of dementia and a history of TBI.

The results of this preliminary study of the TASCI intervention support the feasibility, utility, and acceptance of the intervention. Qualitative findings revealed that caregivers found the program valuable and would recommend it to others. Participating caregivers valued the convenience of the telehealth delivery model, allowing caregivers to be present for care recipients if needed, and appreciated that the program was tailored to their individual needs. Caregivers also reported that TACSI helped them interact with their care recipient more effectively and assisted with their stress management. Some caregivers believed that TACSI would provide even greater benefit to newer caregivers requiring education on AD/ADRD and/or TBI, as well as caregiving.

Quantitative outcomes indicated that TACSI was acceptable, appropriate, and feasible for caregivers. Most caregivers strongly agreed with statements about the delivery and reception of the intervention. Caregiver feedback provided recommendations that were incorporated into the pilot RCT intervention, including the addition of topics related to lifestyle planning and balance, and intimacy.

Similar to our previous study assessing a psychoeducational/psychosocial telehealth intervention for caregivers of cognitively impaired relatives living in residential long-term care (47), caregivers in this study appreciated their coach’s emotional support and reassurance, knowledgeability, and objectivity. The TACSI intervention targeted a different population—caregivers of individuals with both AD/ADRD and TBI who were living in a variety of settings. Our findings are also similar to other studies, including a study examining the outcomes of a telehealth intervention for people living with dementia and their caregivers (48). Their findings indicated that caregivers appreciated an emphasis on overall well-being through lifestyle planning and stress management. Furthermore, caregivers in both interventions noted the importance of flexible and tailored content to meet their educational and emotional needs.

Recent meta-analyses support various nonpharmacological interventions to reduce caregiver burnout and depression and improve quality of life (49,50). The more successful interventions include psychoeducation and multicomponent interventions rather than support groups or respite. A program’s effectiveness largely depends on its focus on matching the caregiver’s needs and its tailoring for specific groups. The semistructured TACSI intervention is an example of 1 such program. Coaches and caregivers jointly selected intervention content from the manual to address each caregiver’s priorities during session discussions. For instance, some caregivers wanted to focus more on stress management while others were interested in how to improve engagement with their care recipient. Some spouses were interested in discussing changes in intimacy and relationship dynamics while these topics were not relevant for adult children. Pertinent topics outside the intervention manual were also welcomed into session discussions. In sum, no 2 caregivers likely received the exact same intervention. The positive outcomes reported here are likely attributable to the program’s tailoring to the caregiver’s unique needs.

### Strengths and Limitations

The study demonstrated several strengths. The multisite trial recruited caregivers from 2 large healthcare organizations. It utilized a recruitment approach based on documented diagnoses of TBI and AD/ADRD, rather than relying on self-report, contributing to the investigation’s validity. The intervention was delivered through telehealth modalities, minimizing caregivers’ travel burden, or finding alternate care for their care recipient. Finally, the study employed a mixed methods approach, collecting and analyzing quantitative and qualitative outcomes to support the results. The qualitative approach gave caregivers a voice in providing feedback for the program, often neglected in dementia caregiver research (51).

Study limitations were also noted. As a feasibility study, the sample size was small and lacked racial and ethnic diversity, limiting the generalizability of results. Future studies will include larger samples with enhanced diversity. Interviewer bias may have occurred due to the caregiver wanting to please the interviewer as a study team member. However, caregivers did share their suggestions for improvements to the program, suggesting they were comfortable sharing constructive feedback.

Some care recipients resided in long-term care, which comes with distinctive challenges and needs that may not apply to all caregivers (52). In addition to addressing their overlapping care needs, their unique caregiving concerns were addressed through the tailored design of TACSI. Additionally, curated residential care content is included in the pilot RCT.

There was variability in the length of time since TBI and dementia diagnoses; therefore, for some caregivers, the need for support may have been less pronounced at the time of intervention receipt. We are continuing to gather data from long-time caregivers and residential caregivers during the pilot RCT, employing the larger sample to further investigate the perceived utility of this intervention for these caregivers.

### Conclusion

The results of this study supported the feasibility, utility, and acceptance of the TACSI intervention for caregivers of people living with TBI and AD/ADRD. Further, they warranted the progression to a pilot RCT of TACSI, which will employ a larger sample size to further assess the feasibility of testing the efficacy of the intervention. Caregivers valued TACSI and would recommend it to others. They appreciated the telehealth delivery model and that the program was personalized to meet their needs. Caregivers also believed TACSI helped manage their stress and improved their interactions with care recipients. Constructive feedback from caregivers included that some felt it would have been more beneficial earlier in their caregiving trajectory and suggested topics for the intervention (lifestyle planning/balance, intimacy, and residential long-term care). For these reasons, further evaluation employing a pilot RCT and a larger, more diverse sample to assess program feasibility and outcomes has begun.

## Supplementary Material

igaf057_suppl_Supplementary_Tables_S1-S4

## Data Availability

We plan for the quantitative data set associated with this paper to be deposited into the Federal Interagency Traumatic Brain Injury Research (FITBIR) Informatics System data repository or DRUM (Data Repository for the University of Minnesota), pending regulations and repository availability at the time of submission. Qualitative data will not be made available.
