# Supplementary material for: The Traumatic Brain Injury-Alzheimer’s Disease and Alzheimer’s Disease-related Dementia Caregiver Support Intervention: A Mixed Methods Evaluation of Program Feasibility, Acceptability, and Utility
Source: Innov Aging. 2025 Jun 18;9(6):igaf057. doi: 10.1093/geroni/igaf057 (PMC12287695; doi:10.1093/geroni/igaf057)
Supplement: igaf057_suppl_Supplementary_Tables_S1-S4 [file igaf057_suppl_supplementary_tables_s1-s4.docx]

***Innovation in Aging* Supplementary Material: Wilson et al. The TBI-AD/ADRD Caregiver Support Intervention: A Mixed Methods Evaluation of Program Feasibility, Acceptability, and Utility.**

**Supplementary Table 1. Intervention Objectives and Key Activities**

| **A. Increase CG understanding of AD and TBI**  1. Provide AD education processes that affect the brain, behavior, and personality  2. Address individual symptoms of AD, providing effective response strategies  3. Provide TBI education: individualized in how TBI affects the brain, behavior, and personality |
| --- |
| **B. Reduce CG Stress**  1. Explain how stress affects the mind and body  2. Increase coping and stress management strategies  3. Introduce Mindfulness Based Stress Reduction (MBSR) strategies and relaxation exercises  4. Encourage self-care |
| **C. Explore and offer support for CG experiences of guilt and grief**  1. Identify, process, and reframe feelings of guilt  2. Explore different grief experiences and their effects on CG and family  3. Explore different types of grief |
| **D. Enhance effective communication**  1. Introduce conflict resolution steps and illustrate how to approach a care issue with medical team  2. Share effective communication strategies  3. Provide coaching to empower CG to initiate and set priorities for care and support  4. Brainstorm how to create and implement care plans |
| **E. Increase CG awareness of AD and TBI supports and services**  1. Provide information on national and local AD supports and services  2. Provide information on national and local TBI supports and services  3. Explore new caregiving roles and responsibilities |
| **F. Enhancing family support**  1. Review context of family caregiving  2. Review effects of AD and TBI on family  3. Explore family communication  4. Encourage family involvement in CR care and support of CG (eg., visits, calls, respite |

Note. CG, Caregiver; AD, Alzheimer’s Disease; TBI, Traumatic Brain Injury; CR, Care Recipient.

**Supplementary Table 2. Baseline Survey Measures**

| **Type of Measure or Outcome** | **Baseline Reliability** | **Description** | **Citation** |
| --- | --- | --- | --- |
| Activities of Daily Living/Instrumental Activities of Daily Living | 0.91 | 12 items measuring the care recipient’s dependence on assistance with ADLs and dependence on assistance with IADLs, on a 3 point scale (no help, some help, a lot of help). | Katz S, Ford AB, Moskowitz RW, Jackson BA, Jaffe MW. Studies of illness in the aged. The Index of ADL: A standardized measure of biological and psychosocial function. JAMA. 1963;185:914-919.  Lawton MP, Brody EM. Assessment of older people: self-maintaining and instrumental activities of daily living. Gerontologist. 1969;9(3):179-186.  Graf C. The Lawton instrumental activities of daily living scale. Am J Nurs. 2008;108(4):52-62; quiz 62-53. |
| Revised-Memory and Behavior Problems Checklist | 0.94 | A list of 24 common behavior and memory problems manifested by people with dementia. Caregivers indicate the presence of behaviors and how much they bothered the caregiver. | Reproduced/revised with permission. Teri L, Truax P, Logsdon R, Uomoto J, Zarit S, Vitaliano PP. Assessment of behavioral problems in dementia: the Revised Memory and Behavior Problems Checklist. Psychol Aging. 1992;7(4):622-631. |
| Modified Caregiver Appraisal Scale | 0.83 | A Likert scale selection of 35 items to measure primary subjective stress and appraisals of primary objective stressors/care demands, specifically in the areas of perceived burden, caregiver relationship satisfaction, caregiving ideology, and caregiving mastery. | Short form: Struchen MA, Atchison TB, Roebuck TM, Caroselli JS, Sander AM. A multidimensional measure of caregiver appraisal: validation of the Caregiver Appraisal Scale in Traumatic Brain Injury. J Head Trauma Rehab 2002;17(2):132-154.  Original scale: Lawton MP, Kleban MH, Moss M, Rovine M, Glicksman A. Measuring caregiving appraisal. J Gerontol 1989; 44: 61-71. |
| Psychological Distress: The Center for Epidemiological Studies-Depression Scale | 0.95 | 20-item measure assessing the number of days participants had experienced depressive symptoms in past week. | Radloff L. The Center for Epidemiological Studies-Depression Scale: A self-report depression scale for research in the general population. Applied Psychological Measurements. 1977;3:385-401. |
| TBI-CareQOL Feelings of Loss – Self – Short Form 6a | 0.90 | 6-item scale assessing feelings of loss, (5 point scale ranging from never to always) | Note modification: Added “illness” to Q2 and Q5.  ©2019-2021 David Cella on behalf of the National Institute for Neurological Disorders and Stroke (NINDS). Some content used with permission of the PROMIS Health Organization. Copyright © December 1, 2016 Noelle E. Carlozzi. All rights reserved. |
| TBI-CareQOL Feelings of Loss – Person with TBI – Short Form 6a | 0.84 | 6-item scale assessing feelings of loss, administered to caregiver about care recipient, (5 point scale ranging from never to always) | Note modification: Added “illness” to Q1 and Q4.  ©2019-2021 David Cella on behalf of the National Institute for Neurological Disorders and Stroke (NINDS). Some content used with permission of the PROMIS Health Organization. Copyright © December 1, 2016 Noelle E. Carlozzi. All rights reserved. |
| TBI-CareQOL Feeling Trapped – Short Form 6a | 0.90 | 6-item scale assessing feeling trapped, (5 point scale ranging from never to always) | Note modification: Added “illness” to Q3 and Q5.  ©2019-2021 David Cella on behalf of the National Institute for Neurological Disorders and Stroke (NINDS). Some content used with permission of the PROMIS Health Organization. Copyright © December 1, 2016 Noelle E. Carlozzi. All rights reserved. |
| TBI-CareQOL Caregiver-Specific Anxiety – Short Form 6a | 0.80 | 6-item scale assessing caregiver anxiety, (5 point scale ranging from never to always) | Note modification: Added “illness” to Q3.  ©2019-2021 David Cella on behalf of the National Institute for Neurological Disorders and Stroke (NINDS). Some content used with permission of the PROMIS Health Organization. Copyright © December 1, 2016 Noelle E. Carlozzi. All rights reserved. |
| TBI-CareQOL Caregiver Strain – Short Form 6a | 0.83 | 6-item scale assessing caregiver strain, (5 point scale ranging from never to always) | Note modification: Added “illness” to Q5 and Q6.  ©2019-2021 David Cella on behalf of the National Institute for Neurological Disorders and Stroke (NINDS). Some content used with permission of the PROMIS Health Organization. Copyright © December 1, 2016 Noelle E. Carlozzi. All rights reserved. |
| TBI-CareQOL Caregiver Vigilance – Short Form 6a | 0.86 | 6-item scale assessing caregiver vigilance, (5 point scale ranging from never to always) | ©2019-2021 David Cella on behalf of the National Institute for Neurological Disorders and Stroke (NINDS). Some content used with permission of the PROMIS Health Organization. Copyright © November 6, 2017 Noelle E. Carlozzi. All rights reserved. |
| TBI-CareQOL Emotional Suppression – Short Form 6a | 0.89 | 6-item scale assessing emotional suppression, (5 point scale ranging from never to always) | ©2019-2021 David Cella on behalf of the National Institute for Neurological Disorders and Stroke (NINDS). Some content used with permission of the PROMIS Health Organization. Copyright © November 6, 2017 Noelle E. Carlozzi. All rights reserved. |
| TBI-CareQOL Family Disruption – Short Form 3a | 0.61 | 3-item scale assessing family disruption, (5 point scale ranging from never to always) | Note modification: Added “illness” to Q1 and Q2.  Copyright © November 6, 2017 Noelle E. Carlozzi. All rights reserved. |
| COPE | 0.67 | A selection of 24 items from three domains of COPE (problem-focused coping, emotion-focused coping, and avoidant coping) assessing the coping style of caregivers to stressors related to care provision. Response options range 1 (‘I usually don’t do this at all’) to 4 (‘I usually do this a lot’) | Original: Carver, C. S., Scheier, M. F., & Weintraub, J. K.  (1989).  Assessing coping strategies:  A theoretically based approach.   Journal of Personality and Social Psychology, 56, 267-283. Instructions, select questions, & response items from: Carver, C. S. (2013). COPE Inventory. Measurement Instrument Database for the Social Science. |
| Interpersonal Support Evaluation List | 0.86 | A 16 item short version of the scale measuring the caregiver’s perception of social support, where respondent indicates ‘definitely true’, probably true, probably false, definitely false’ | Shortened scale: Payne, Thomas J, Andrew, Michael, Butler, Kenneth R, Wyatt, Sharon B, Dubbert, Patricia M, & Mosley, Thomas H. (2012). Psychometric Evaluation of the Interpersonal Support Evaluation List–Short Form in the ARIC Study Cohort. SAGE Open, 2(3), 1-8.  Original/full scale: Cohen, S., Mermelstein, R., Kamarck, T., & Hoberman, H. (1985). Measuring the functional components of social support. In I. G. Sarason & B. R. Sarason (Eds.), Social support: Theory, research and application (pp. 73-94). The Hague, The Netherlands: Martinus Nijhoff. Instructions: https://www.midss.org/content/interpersonal-support-evaluation-list-isel |
| Caregiver Self-Efficacy | 0.75 | 8-item measure assessing caregiver self-efficacy of completing caregiver tasks. Responses ranged from 1 (not certain at all) to 10 (very certain). | Fortinsky RH, Kercher K, Burant CJ. Measurement and correlates of family caregiver self-efficacy for managing dementia. Aging Ment Health. 2002;6(2):153-160. |
| Community-Based Service Use | 0.85 | Family member indicates if care recipient utilized any of 14 formal/paid home and community-based services over the past 3 months, with an additional item serving as a write-in response. | Concepts adapted from:  Amjad H, Wong SK, Roth DL, et al. Health services utilization in older adults with dementia receiving care coordination: the MIND at home trial. *Health Serv Res.* 2018;53(1):556-579.  Mittelman MSEC, Pierzchala A. Counseling the Alzheimer's caregiver: A resource for health care professionals. Chicago: AMA Press; 2003. |

Note. Baseline reliability was assessed using Cronbach’s alpha.

**Supplementary Table 3. Semi-Structured Interview Guide Questions**

| **Introduction** |
| --- |
| My objective in this interview is to determine whether and how TACSI program benefited or did not benefit you and your <relative>/person you care for.  1. Just to get us started, can you tell me a little bit more about your relative.  2. When did their TBI occur? Did they have more than one? (If yes,) When did they occur?  3. When did you first notice their dementia/memory problems?  4. When did you first start providing help to your relative because of her/his dementia or TBI?  5. What type of help or care do you provide to your relative?  6. How are things going now for you and your relative? |
| **Enrollment reason(s)** |
| 7. Why did you decide to enroll in this project? |
| **Overall Impression** |
| 8. What was your overall impression of the TACSI program? |
| **Harm and Drawbacks** |
| 9. What negative outcomes, if any, resulted from being involved in the TACSI program for you? Why (or how) do you think that/these happened?  10. What negative outcomes, if any, resulted from the TACSI program for your relative? Why (or how) do you think that/these happened? |
| **Benefits** |
| 11. What benefits, if any, resulted from involvement in the TACSI program for you? Why (or how) do you think that/these happened?  12. What benefits, if any, resulted from involvement in the TACSI program for your relative? Why (or how) do you think that/these happened?  13. Do you feel like your use of TACSI had any effect on your: a) interactions with your relative? b) interactions with other family members besides your relative? c) interactions with your relative’s healthcare team or facility staff (if applicable)? |
| **TACSI Components** |
| 14. I’d like to walk through the various service components of the TACSI program and ask how each component did or did not help you or your relative and why:  a) Which individual coaching sessions or topics helped most? Least? Why?  b) Did you have any family members participate in a session along with you? <if yes> What happened in the family sessions that helped the most? And the least? Why?  c) How did discussing your relative’s care with <coach> help or not help? <i.e. make adjustments to relative’s care, have subsequent discussions with healthcare team or family members>  d) What parts of TACSI do you think should be changed, added, or removed? Why? |
| **Links to Outcomes** |
| 15. Do you feel like your involvement in TACSI had any effect on your feelings of stress and burden?  16. Do you feel like your involvement in the TACSI program had any effect on your ability to care for and engage with your relative?  17. Do you feel like your involvement in the TACSI program had any effect on your mood?  *VA participant only questions:*  18a. Have you participated in any VA caregiver support programs (like the VA program examples)?  18b. Do you participate in the VA caregiving stipend program (i.e. VA examples)?  18c. If yes to a or b: How does the TACSI program compare to those other VA caregiver support programs? |
| **Ending Questions** |
| 19. Thank you for helping us learn more about your experiences with the TACSI program. Is there anything else you’d like to share with me today? |

Note. TACSI, Traumatic Brain Injury - Alzheimer’s Disease and Alzheimer’s Disease Related Dementias Caregiver Support Intervention; TBI, Traumatic Brain Injury; VA, Veterans Affairs.

**Supplementary Table 4. Caregiver and Intervention Participation Characteristics**

| **Pseudonym** | **Caregiving Duration (years)** | **Intervention Session Modality** | **Intervention Session Average Duration (mins)** | **Ad Hoc Support Modality** | **Ad Hoc Session Average Duration (mins)** |
| --- | --- | --- | --- | --- | --- |
| *Spouse* |  |  |  |  |  |
| Mary | 6 | phone, video conferencing | 48 | email |  |
| Meryl | 43 | phone | 50 | email |  |
| Linda | 2 | video conferencing | 65 | email |  |
| Pam | 3 | phone | 62 | phone | 35 |
| Shannon | 10 | phone | 58 | none |  |
| Greta | 8 | video conferencing | 62 | video conferencing | 15 |
| Peggy | 51 | phone | 53 | none |  |
| Helen | 1 | phone, video conferencing | 64 | email |  |
| Edie | 25 | phone | 45 | email |  |
| *Adult Child* |  |  |  |  |  |
| Sam | 1 | video conferencing | 63 | email |  |
| Elena | 8 | phone | 66 | phone | 40 |
| Chris | 3 | video conferencing | 65 | email |  |
| Angela | 8 | phone, video conferencing | 63 | email |  |
| Becky | 6 | video conferencing | 56 | email |  |
| Simone | 17 | phone | 62 | email |  |
